# Analysis of the Dissipation Behavior of Defoliants in Cotton Fiber during Field and Scouring Process Using Liquid and Gas Chromatography

**DOI:** 10.1155/2019/2879074

**Published:** 2019-10-30

**Authors:** Mingbo Ma, Pirah Ayaz, Wanhui Jin, Munir Hussain, Wenlong Zhou

**Affiliations:** ^1^Department of Textile Materials, Zhejiang Sci-Tech University, Hangzhou, Zhejiang 310018, China; ^2^Hubei Province Fibre Inspection Bureau, Wuhan, Hubei 430000, China; ^3^Department of Polymer Science and Engineering, Hangzhou, Zhejiang 310018, China

## Abstract

Defoliants carried by cotton fiber could harm production workers and consumers through respiratory and dermal exposure. This study was carried out to evaluate the dissipation behavior of four commonly used defoliants tribufos, diuron, thidiazuron, and ethephon in cotton fiber during field stage and also in cotton scouring using liquid chromatography and gas chromatography. Field trials showed that although all the defoliants dissipated fast, however, the fiber from the tribufos and ethephon applied field had considerable potential to exceed the maximum residue limit when the fiber was harvested at common intervals after application of defoliants. The defoliant residues could be removed completely from the defoliant-carrying cotton textiles during alkaline scouring. The results indicated that attention should be paid to the risk of occupational exposure to these defoliants rather than consumer exposure. Fiber harvest on the tribufos and ethephon applied fields is recommended after a 1-week delay in order to reduce the residues to an acceptable level.

## 1. Introduction

Cotton fiber is one of the most important raw materials for the textile industry and occupies more than 40% of the global total textile fiber consumption for a long time. As estimated, a global yield of 24.6 million tons of cotton fiber was produced on about 32.3 million hectares of cotton fields in crop-year 2017, and almost half of the fiber was harvested by machine [1, 2]. Cotton fiber is easily contaminated by dry leaf fragments during machine picking and reduces the quality grade of the fiber. Defoliants, therefore, are applied on cotton plants previously in order to promote leaf abscission. In the world, the most widely used active ingredients of defoliants are diuron, thidiazuron, tribufos, and ethephon (structure and physicochemical properties shown in Table 1), which were applied at an average application rate of 1 kg/ha [1].

However, massive use of these cotton defoliants has caused an environmental concern and public health problems due to their relatively high toxicity. For example, tribufos shows an oral median lethal dose (LD_50_) of 150 mg·kg^−1^ in rats and is considered to be highly hazardous [2]. Furthermore, most of the degradation products of these defoliants exhibit much higher toxicity and are persistent in the environment [3–5]. There are considerable reports concerning their acute and chronic risks to the terrestrial and aquatic life nearby the applied cotton fields [6, 7]. Defoliant application also leads to safety problems to cotton-related agricultural and sideline products, such as cottonseed oil and honey, in which defoliant residues have been frequently detected [8, 9]. Furthermore, a report indicated that people living in the communities near defoliant-applied cotton fields are 60–100% more frequently infected by respiratory and irritation disease than the residents in non-cotton-growing agricultural communities [10].

Defoliants tribufos, diuron, thidiazuron, and ethephon are chemically stable and can persist for long periods in soil and water [11–14]. They were usually applied at about 2 weeks before the fiber harvest [15]. As a result, raw cotton fiber usually carries a considerable amount of defoliants. We inspected 21 of machine-harvested cotton samples from the market, and the results show that they all carried defoliants, with content varying from 0.01 to 9.78 mg/kg (data not published). The amount of defoliants in 9 of these samples exceeds the limit value for organic agricultural chemicals in textiles (5 mg/Kg) according to the Oeko-Tex® Standard 100. The potential risk of the cotton fiber from defoliant-applied fields cannot be ignored. Defoliants-carrying fiber may harm workers in cotton mills and consumers via respiratory and dermal exposure, and the extent of the risk is highly depended on the amount of defoliants in the fiber. Unfortunately, there is little information about the dissipation of the defoliant in cotton fiber during cultivating and processing stage. In this study, field experiment was performed to evaluate the dissipation behavior of the four widely used defoliants in the fiber during the growing period. The effects of the main chemical processing for cotton products, i.e., scouring, on the dissipation of the defoliants were also analyzed.

## 2. Methods

### 2.1. Chemicals and Reagents

Defoliant tribufos, diuron, thidiazuron, and ethephon standards (>97% purity) were supplied by Aladdin Chemistry Co., Ltd. (Shanghai, China). Commercial wettable powder formulations of these defoliants, containing 30%, 35%, 50%, and 40% of the active ingredient, respectively, were purchased from DuPont Agricultural Chemicals Ltd. (Shanghai, China). A fatty alcohol polyoxyethylene ether surfactant JFC-6 and analytical grade sodium hydroxide were both from Hangzhou Mike Chemical Instrument Co., Ltd. (Hangzhou, China).

### 2.2. Field Experiments

The field experiment was carried out at Zhejiang Academy of Agricultural Sciences (120.2°E, 30.3°N, Zhejiang, China; temperature 28–37°C and no rain during the study period) in Oct. 16–Nov. 13, 2013. Cotton (variety GK39) was planted in sandy loam soil. When about 70% of the cotton bolls opened, the opened bolls were marked before defoliant application. The defoliant tribufos, diuron, thidiazuron, and ethephon formulations (containing 30%, 35%, 50%, and 40% of the active ingredient) were mixed with water at a volume ratio of 1 : 1000 in an artificial pesticide-spraying vehicle. The defoliant solutions were then applied on cotton at normal rates, i.e., 1.26, 0.84, 0.42, and 2.24 Kg/ha, respectively. Fiber samples were taken every 7 days for quantification analysis of the carried defoliants.

### 2.3. Extraction of Defoliants

The extraction solvent for diuron and thidiazuron was both acetonitrile and those for tribufos and ethephon were methanol and acidic methanol (2% v/v acetic acid in methanol), respectively. Cotton fiber (15.000 g) was mixed with the extraction solvent at a solid-to-liquor ratio of 1 : 35 (g/mL), and then ultrasoniced in an ultrasonic bath at 30°C for 90 mins. The liquid was wrung out of the fiber and then filtered through membrane filters (pore size 0.45 *μ*m). Each fiber sample was extracted 3 times in order to exhaustively extract the defoliants. The extracts of each defoliant were combined and concentrated under vacuum to a small volume. Finally, the concentrated extract was diluted to the mark with methanol in a 10 mL volumetric flask and used for quantification analysis.

### 2.4. Quantification Analysis

The diuron and thidiazuron extracts were analyzed by using an Agilent 1260 high-performance liquid chromatography (HPLC) system equipped with a 1260 variable wavelength UV detector. Agilent SB C18 (4.6 × 150 mm, 3.5 *μ*m) was used at 30°C, the injection volumes for all the samples were 20 *μ*L, and elution flow rates were 1.0 mL/min. The mobile phase for analysis of diuron and thidiazuron was 60% and 50% aqueous methanol, and the detection wavelength was 250 and 280 nm, respectively.

Analysis of the tribufos and ethephon extracts was performed on an Agilent 6890N gas chromatography (GC) apparatus equipped with an autoinjector split 50 : 1 onto a DB-5MS column (30 m × 0.25 mm, 0.25 *μ*m, J&W Scientific). The temperature condition for analysis of tribufos is programmed from 180°C to 280°C at 7°C·min^−1^, while for analysis of ethephon, the temperature was from 125°C to 250°C at 10°C·min^−1^. The injection volumes were both 2 *μ*L, and injection temperature was 250°C with the split-less mode. The flow rate of carrier gas (N_2_) was 1.0 mL/min.

### 2.5. Validation of the Quantification Methods

Quantification of tribufos, diuron, thidiazuron, and ethephon was previously created based on their standard chemicals (>97% purity) and chromatography methods. The analytical parameters of the quantification methods were evaluated according to Commission Regulation EC No. 333/2007 and other publications [16], including linearity, limit of detection (LOD), limit of quantification (LOQ), linearity, and recovery.

### 2.6. Sample Scouring

Scouring of the cotton samples was performed in a conventional boiling alkaline bath consisting of 20.0 g/L aqueous sodium hydroxide and 4.0 g/L JFC-6 surfactant, as we previously described [17]. Degradation behavior of the defoliant standards in the scouring bath was also investigated; 2 mL of 1.0 g/L methanolic pure defoliant solutions were added into 98 mL of the boiling scouring bath. At each time interval, 20 mL of the mixture solution was taken out and neutralized with 0.5 M chlorhydric acid. After being concentrated and diluted to mark, the solutions were analyzed by quantification methods described above.

## 3. Results and Discussion

### 3.1. Validation of the Analytical Methods

The validation of the HPLC method for the determination of diuron and thidiazuron and the GC method for tribufos and ethephon showed that the quantification methods provided satisfactory performance. The main results are shown in Table 2. The chromatography method used for each defoliant was the optimized one, and no interferences were observed in the retention times of the compounds analyzed. Linearity was evaluated using the matrix-matched method, with solutions prepared at seven analyte concentration levels. The correlation coefficients (*R*^2^) obtained by linear regression analysis were greater than 0.99, indicating the linear regression equations could be employed. The four analytes showed recoveries in the range from 87.5 to 93.1%. The LOD and LOQ were in the range from 7 to 25 *μ*g/L and from 16 to 60 *μ*g/Kg, respectively. The LOQ for the four defoliants all meets the LOQ requirements (0.5 mg/Kg) of Oeko-Tex® Standard 100 toward agriculture chemicals in textiles. These results indicated that the established HPLC and GC methods could be used for quantification of the defoliants in cotton fiber.

### 3.2. Dissipation Behavior of Defoliants during Field Stage

The amount of defoliants carried by newly harvested cotton fiber highly depends on the time interval from the defoliant application. Knowing the rate of dissipation of a defoliant in cotton fiber is important in understanding its potential health risks to workers and consumers. As shown in Table 3, defoliants tribufos, diuron, thidiazuron, and ethephon in the fiber from the bolls opened prior to defoliant application all dissipated rather fastly in two weeks under field conditions. Their concentration in fiber had dropped to 4.8, 3.3, 1.3, and 6.7 mg/Kg, with reduction rates of 61.3%, 68.3%, 66.7%, and 65.6% of their initial concentrations. Surprisedly, the fiber from the newly opened bolls was also found to carry defoliants, with concentrations 2.6–5.2 times lower than those in the fiber from the previously opened bolls. This could probably be attributed to transfer the defoliants from the surface of the cotton bolls to the inside fiber and/or from the surrounding environment through adsorption. Cotton fiber is usually harvested about 2 weeks after the defoliant is applied. More attention should be paid to the newly harvested fiber applied with tribufos and ethephon because of the relatively high concentration of residues. According to the limits to organic agricultural chemicals in textiles proposed by Oeko-Tex® Standard 100 (5 mg/Kg), the fiber from tribufos and ethephon applied field has considerable potential to exceed the maximum levels.

The amount of residues progressively decreased when the harvest delayed, with only 15.5%, 12.0%, 9.4%, and 16.9% of their initial concentrations detected at 28-day time point. According to the dissipation behavior of the four defoliants obtained above, fiber from tribufos and ethephon applied field are recommended to be harvested later than the common harvest proceeds.

### 3.3. Effects of Scouring

Alkaline scouring is the first stage during the wet processing of cotton textiles, aiming at removal of the surface noncellulose impurities and improving their dyeability and comfortability. Therefore, the effect of scouring on the dissipation of the defoliants in cotton fiber is investigated. Fortunately, no defoliant residues in the fiber were detected after scouring. They probably transferred to the scouring bath, accompanied by the removal of the impurity layer from the fiber surface.

**Figure 1:**
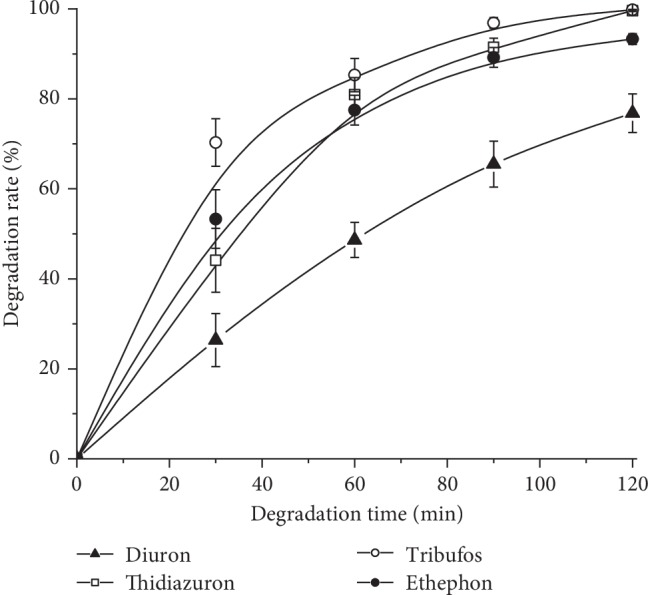
Kinetic degradation of defoliant standard in the scouring bath.

These defoliants are extremely stable in the neutral solutions at room temperatures [1]. It is necessary to investigate the dissipation of the defoliants in the scouring bath, characterized by concentrated alkali and high temperature (about 100°C). This facilitates to evaluate their environmental effects after the scouring waste being discharged.  Figure 1 shows the degradation behavior of the defoliants in boiling scouring bath. It is clearly seen that tribufos degrades most fastly and ethephon, thidiazuron, and diuron are in the following order. The dissipation was further analyzed using a well-known hyperbolic rate model (equations (1) and (2)) [16] based on the residue data obtained at selected time intervals:

(1) $$\begin{array}{c} C=C_{0}e^{-kt}, \end{array}$$

(2) $$\begin{array}{c} \ln\left(\frac{C_{0}}{C}\right)=kt, \end{array}$$

(3) $$\begin{array}{c} t_{1/2}=\frac{1}{k}\ln\;2, \end{array}$$

where *C* is the concentration of defoliants at time *t* in the scouring bath; *C*_0_ = C (time 0); *k* is the degradation rate constant (min^−1^); and *t*_1/2_ is the degradation half-time of the defoliants in the scouring bath.

The kinetic results are shown in Table 4. The determination coefficients (*R*^2^) obtained were greater than 0.975, indicating that the fits of the models could be employed and the kinetic dissipation of the four defoliants could be predicted. Unlike the dissipation behavior in moderate conditions, the four defoliants all decomposed very quickly in the boiling scouring bath. The dissipation half-time (*t*_1/2_) for tribufos, diuron, thidiazuron, and ethephon were only 20.4, 69.0, 24.5, and 23.8 mins, which were much lower than the time for scouring of commercial cotton textiles (about 2 hours). Therefore, it can be concluded that the defoliant tribufos, diuron, thidiazuron, and ethephon carried by cotton fiber could be decomposed completely in the scouring bath.

The compositions of the degradation products of these defoliants in the scouring bath were not investigated in this study. However, this work should be encouraged because the degradation products may be highly toxic and may harm the environment when they are discharged with the scouring wastes.

## 4. Conclusions

Field trials showed that although all the defoliants dissipated fast, however, if the fiber from the tribufos and ethephon applied field is harvested at common intervals after application of defoliants, i.e., two weeks, the fiber has considerable potential to exceed the maximum residue limit as proposed by Oeko-Tex® Standard 100 (5 mg/Kg). The defoliant residues could be removed completely from the defoliant-carrying cotton textiles during alkaline scouring. The results indicated that attention should be paid to the risk of occupational exposure to these defoliants rather than consumer exposure. Inspection and supervision toward the defoliants in raw cotton fiber are necessary although these agriculture chemicals are currently ignored.

## Figures and Tables

**Table 1:** Physicochemical properties of the defoliants tribufos, diuron, thidiazuron, and ethephon.

Defoliants	Chemical structure	Vapor pressure (mm Hg, 25°C)	Half-life in field soil (days)	Hydrolysis half-life (days)	Toxicity^a^
Tribufos	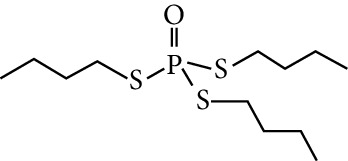	6.50 × 10^−6^	64.8	124 (pH 9)	192
Diuron	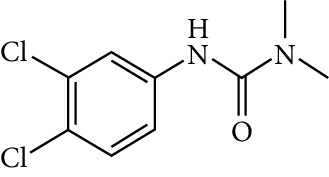	6.90 × 10^−8^	99.9–134	1240–1330 (pH 7)	500
Thidiazuron	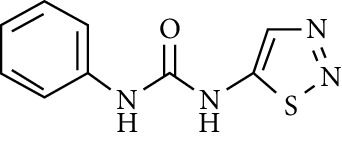	2.30 × 10^−11^	11.1–16.8	337 (pH 7)	3740
Ethephon	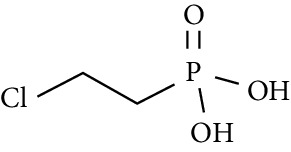	9.80 × 10^−8^	3.3–9.7	—	3122

^a^Expressed as oral LC_50_ in rats (mg/Kg).

**Table 2:** Figures of merit for the analytical methods.

Compound	Retention time (min)	Equation of regression (mg/L), *R*^2^	Linear range (mg/L)	LOD/LOQ (*μ*g/L, *μ*g/Kg)	Recovery (*n* = 5)
Tribufos	8.63	*y* = 181719*x* − 1107900, *R*^2^ = 0.9949	0.1–200	12/30	93 ± 6
Diuron	6.46	*y* = 85.573*x* + 4.785, *R*^2^ = 0.9998	0.1–50	10/50	89 ± 6
Thidiazuron	6.72	*y* = 7795*x* + 1977, *R*^2^ = 0.9996	0.1–100	25/60	87 ± 5
Ethephon	7.27	*y* = 535.43*x* − 4.964, *R*^2^ = 0.9997	0.1–125	7/16	91 ± 4

**Table 3:** Defoliant residues in cotton fiber after different time intervals from their application on the cotton field.

Time from application		Tribufos^a^ (mg/kg)		Diuron (mg/kg)		Thidiazuron (mg/kg)		Ethephon (mg/kg)	
		Opened^b^	Unopened^c^	Opened	Unopened	Opened	Unopened	Opened	Unopened
1 hour		12.4 ± 1.1	ND	10.2 ± 0.8	ND	5.3 ± 0.4	ND	19.5 ± 1.3	ND
7 days		7.6 ± 0.5	2.5 ± 0.2	5.3 ± 0.7	1.5 ± 0.2	2.5 ± 0.2	0.9 ± 0.1	11.8 ± 1.0	2.2 ± 0.3
14 days		4.8 ± 0.3	1.5 ± 0.2	3.3 ± 0.3	1.0 ± 0.2	1.3 ± 0.1	0.5 ± 0.1	6.7 ± 0.9	1.3 ± 0.2
21 days		3.4 ± 0.3	1.0 ± 0.1	2.2 ± 0.3	0.8 ± 0.1	0.7 ± 0.1	0.3 ± 0.0	4.5 ± 0.6	0.8 ± 0.1
28 days		1.9 ± 0.2	0.5 ± 0.1	1.3 ± 0.2	0.4 ± 0.1	0.5 ± 0.1	0.1 ± 0.0	3.3 ± 0.5	0.5 ± 0.1

^a^Results are expressed as the mean ± standard deviation of three replicate samples. ^b^Fiber from cotton bolls opened prior to pesticide application. ^c^Fiber from cotton bolls opened posterior to pesticide application. ND: not detected.

**Table 4:** Parameters of kinetic degradation of defoliants in the alkaline scouring bath.

Compound		*R* ^2^	*k* (min^−1^)	*t*_1/2_ (min)
Tribufos	ln *C*/*C*_0_ = −0.0339*t* + 0.5257	0.9757	0.0339	20.4
Diuron	ln *C*/*C*_0_ = −0.0317*t* + 2.9969	0.9965	0.0317	69.0
Thidiazuron	ln *C*/*C*_0_ = −0.0283*t* + 5.1153	0.9942	0.0283	24.5
Ethephon	ln *C*/*C*_0_ = −0.0291*t* + 5.1153	0.9953	0.0301	23.8

## Data Availability

The data used to support the findings of this study are available from the corresponding author upon request.
